# Captopril, a Renin-Angiotensin System Inhibitor, Attenuates Features of Tumor Invasion and Down-Regulates C-Myc Expression in a Mouse Model of Colorectal Cancer Liver Metastasis

**DOI:** 10.3390/cancers13112734

**Published:** 2021-05-31

**Authors:** Georgina E. Riddiough, Theodora Fifis, Katrina A. Walsh, Vijayaragavan Muralidharan, Christopher Christophi, Bang M. Tran, Elizabeth Vincan, Marcos V. Perini

**Affiliations:** 1 Department of Surgery, Austin Health Precinct, The University of Melbourne, Austin Health, Lance Townsend Building, Level 8, 145 Studley Road, Heidelberg, VIC 3084, Australia; georgina.riddiough@unimelb.edu.au (G.E.R.); tfifis@unimelb.edu.au (T.F.); kawalsh@unimelb.edu.au (K.A.W.); v.muralidharan@unimelb.edu.au (V.M.); c.christophi@unimelb.edu.au (C.C.); 2 Department of Infectious Diseases, The Peter Doherty Institute, The University of Melbourne, Melbourne, VIC 3000, Australia; manht@unimelb.edu.au; 3 Victorian Infectious Diseases Reference Laboratory, The Peter Doherty Institute, Melbourne, VIC 3000, Australia; 4 Curtin Medical School, Curtin University, Perth, WA 6102, Australia

**Keywords:** renin-angiotensin system, liver regeneration, Wnt pathway, c-myc, colorectal liver metastasis

## Abstract

**Simple Summary:**

Approximately 25% of patients with colorectal cancer will present with or develop colorectal liver metastasis (CRLM). Surgical resection of CRLM offers these patients the best chance of a cure. However, liver resection and the subsequent regenerative response has been linked to tumor recurrence in the liver remnant. The Wnt/β-catenin pathway is one of many pathways common to both post-hepatectomy liver regeneration and tumorigenesis. Wnt signaling modulates multiple genes of the renin-angiotensin system (RAS), and Wnt inhibition can attenuate fibrotic responses and improve cancer outcomes via diverse mechanisms. In this study, we investigate the effects of captopril, a RAS inhibitor (RASi), on the Wnt/β-catenin pathway and phenotypic changes associated with tumor progression in the context of the regenerating liver. We show that RASi induced increased Wnt signaling whilst downregulating features of epithelial-to-mesenchymal transition (EMT). Furthermore, RASi induced significant down-regulation of Wnt target genes, c-myc and cyclin D1, indicating that expression of these genes can be down-regulated by RASi despite the accumulation of stabilized β-catenin.

**Abstract:**

(1) Background: Recent clinical and experimental data suggests that the liver’s regenerative response following partial hepatectomy can stimulate tumor recurrence in the liver remnant. The Wnt/β-catenin pathway plays important roles in both colorectal cancer carcinogenesis and liver regeneration. Studies have shown that the Wnt/β-catenin pathway regulates multiple renin-angiotensin system (RAS) genes, whilst RAS inhibition (RASi) reduces tumor burden and progression. This study explores whether RASi attenuates features of tumor progression in the regenerating liver post-hepatectomy by modulating Wnt/β-catenin signaling. (2) Methods: Male CBA mice underwent CRLM induction, followed one week later by 70% partial hepatectomy. Mice were treated daily with captopril, a RASi, at 250 mg/kg/day or vehicle control from experimental Day 4. Tumor and liver samples were analyzed for RAS and Wnt signaling markers using qRT-PCR and immunohistochemistry. (3) Results: Treatment with captopril reduced the expression of down-stream Wnt target genes, including a significant reduction in both c-myc and cyclin-D1, despite activating Wnt signaling. This was a tumor-specific response that was not elicited in corresponding liver samples. (4) Conclusions: We report for the first time decreased c-myc expression in colorectal tumors following RASi treatment in vivo. Decreased c-myc expression was accompanied by an attenuated invasive phenotype, despite increased Wnt signaling.

## 1. Introduction

Around 25% of patients with colorectal cancer (CRC) will present with or develop colorectal liver metastasis [1]. Surgical resection of colorectal liver metastases offers eligible patients the best chance of a cure. When combined with neo-adjuvant chemotherapy, liver resection for colorectal liver metastasis (CRLM) can achieve 5-year survival rates of almost 25% [2]. However, clinical and experimental data suggests that liver resection, and the subsequent restoration of liver volume and function brought about via a regenerative response, stimulates tumor progression in the liver remnant [3,4]. It is postulated that tumor recurrence in the regenerating liver may occur due to the presence of small, residual tumor deposits that could not be identified pre-operatively, or due to the possible reactivation of dormant cancer stem cells present in the residual liver at the time of resection [5].

The Wnt/β-catenin pathway is one of many pathways common to both post-hepatectomy liver regeneration and tumorigenesis. Wnt signaling is an evolutionary conserved pathway which regulates cell fate, migration, polarity and organogenesis. In health, the canonical Wnt pathway controls the degradation of β-catenin, which in turn influences the transcription of Wnt target genes in a context-dependent manner [6,7]. Wnt signaling plays a central role in liver regeneration and is activated rapidly following partial hepatectomy. Nuclear β-catenin levels rise 2.5-fold in hepatocytes during liver regeneration following partial hepatectomy and this promotes the transcription of Wnt target genes, including the cell cycle regulator cyclin D1 and c-myc [8,9].

Dysregulation of the canonical Wnt pathway occurs in upwards of 80% of CRC cases [10,11]. In many cases of CRC, an inherited or acquired mutation of the *Adenoma Polyposis Coli* (*APC*) gene leads to the formation of a faulty degradation complex, which inadequately degrades β-catenin. Subsequently, the transcriptional effects of β-catenin remain unchecked and Wnt/β-catenin signaling is constitutively turned on [12,13]. Wnt signaling has also been identified as a key regulator of epithelial-to-mesenchymal transition (EMT) [14] and the reverse, mesenchymal-to-epithelial transition (MET) [15]. Notably, Wnt/β-catenin signaling is regulated during EMT and MET in CRC, despite mutations to *APC* [15,16,17,18]. Moreover, in CRC, Wnts and their Frizzled (FZD) receptors are over-expressed, and Wnt/FZD inhibitors have demonstrated anti-tumor effects in CRC [19,20,21,22]. This indicates complex regulation of Wnt signaling in colon cancer and variable signaling dictated by the tumor microenvironment.

The role of the renin-angiotensin system (RAS) in liver and renal fibrosis has been extensively explored. It has been shown in inflammatory conditions such as hepatic and renal fibrosis that angiotensin II (ANGII), the primary effector of the RAS, activates TGFβ1 and NF-kB signaling pathways [23]. Studies in renal fibrosis have shown that the Wnt/β-catenin pathway regulates multiple RAS genes in renal tissue. Furthermore, a small molecule inhibitor of Wnt signaling (ICG-001) was able to attenuate renal fibrosis [24,25]. This is partly due to the role of Wnt signaling in EMT. Wnt signaling inhibition suppresses EMT, leading to diminished replacement of healthy renal tissue by mesenchymal fibroblastic cells [24]. Additionally, inhibition of RAS was associated with a reduction in cancer cell migration and reduced Zeb-1 expression in vitro [26]. Collectively, there is growing molecular and functional evidence that inhibitors of the classical RAS reduce tumor growth and progression and might augment cancer therapies [27,28,29,30].

We have previously shown that RASi could significantly reduce tumor burden and tumor progression in a mouse model of colorectal liver metastasis [31] and that this occurs, at least in part, via immunomodulatory mechanisms [28]. Considering the links between the RAS, Wnt/β-catenin pathway and cancer, in this study we investigated whether RAS inhibition by captopril could modulate the Wnt/β-catenin pathway and EMT/MET in our mouse model of colorectal cancer liver metastasis in the context of liver regeneration following partial hepatectomy.

## 2. Results

### 2.1. Captopril Treatment was Associated with a Significant Down-Regulation of c-Myc Expression

**Figure 1:**
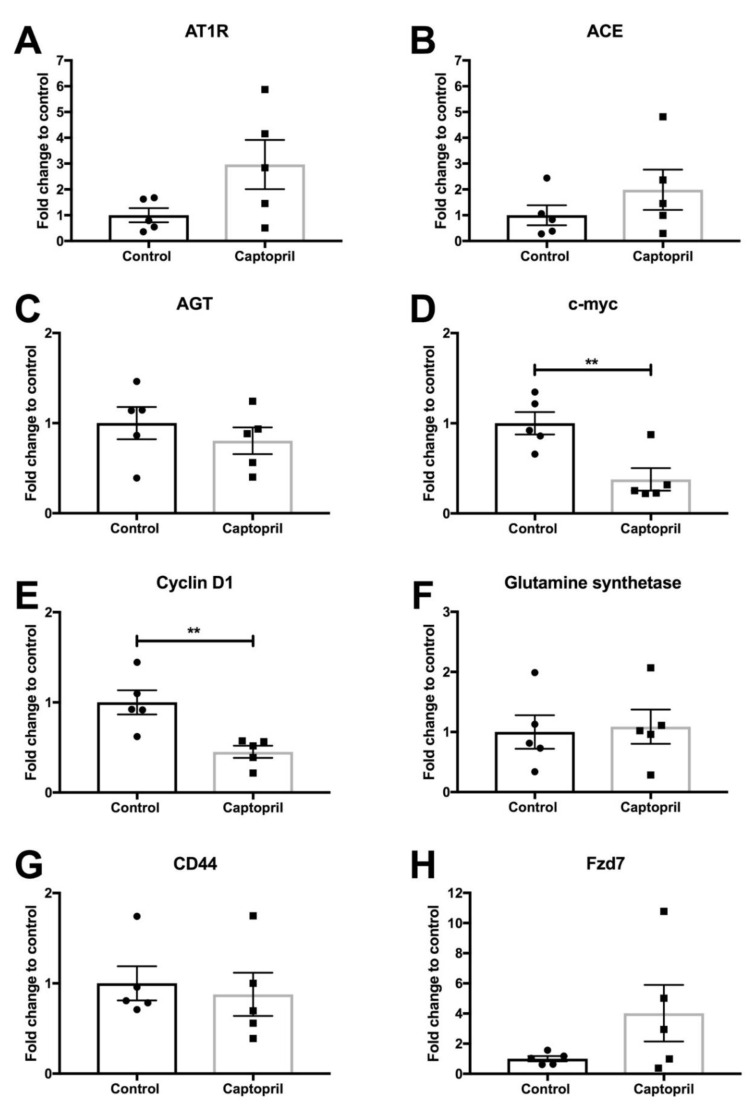
Gene expression (qRT-PCR) in tumor samples. Tumor tissues from control and captopril treated mice were analyzed by qRT-PCR for the expression of the indicated genes. Captopril treatment was not associated with any significant changes in (**A**) AT1R (*p* = 0.0833), (**B**) ACE (*p* = 0.2916), (**C**) AGT (*p* = 0.4245, (**F**) glutamine synthetase (*p* = 0.8281, (**G**) CD44 (*p* = 0.7008 or (**H**) Fzd7 (*p* = 0.1478) expression. Captopril treatment was associated with a significant reduction in (**D**) c-myc (*p* = 0.0078) and (**E**) cyclin-D1 (*p* = 0.0066) expression. Unpaired, two tailed *t* test. ** *p* < 0.01 (*n* = 5 mice).

**Figure 2:**
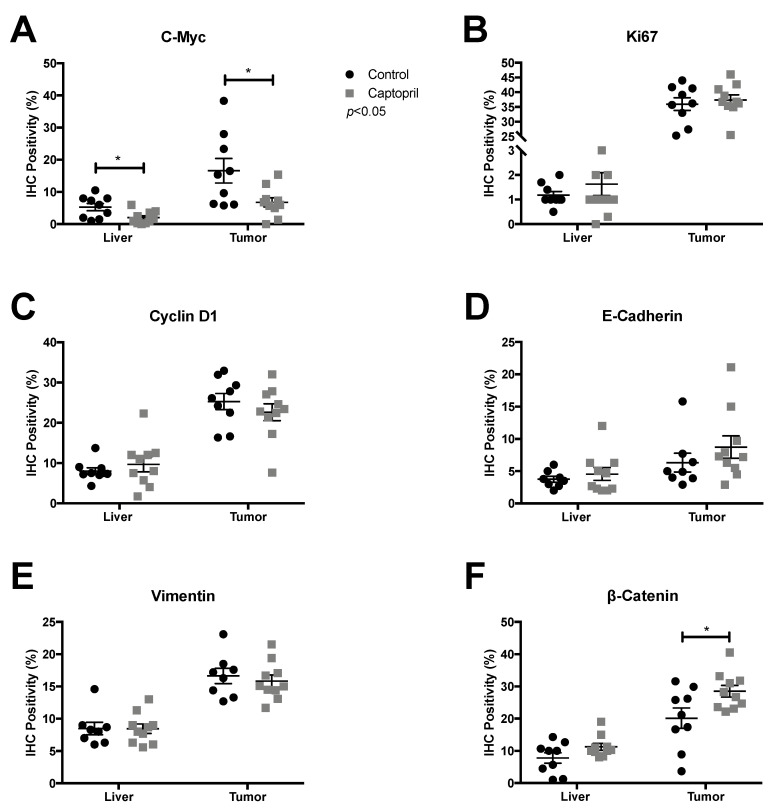
IHC on tissues from captopril treated and control mice. Fixed tissue sections (control, *n* = 9 and captopril, *n* = 10) were stained for the indicated proteins. Captopril treatment was associated with a significant reduction of (**A**) c-myc IHC detection in both the liver (*p* = 0.0185) and tumor (*p* = 0.0218) and a significant increase in (**F**) β-catenin IHC detection within tumor (*p* = 0.0302). Captopril was not associated with any significant changes in (**B**) Ki67 IHC detection for either liver (*p* = 0.3897) or tumor (*p* = 0.6071), (**C**) Cyclin D1 IHC detection for either liver (*p* = 0.4397) or tumor (*p* = 0.3713), (**D**) E-Cadherin IHC detection for either liver (*p* = 0.5041) or tumor (*p* = 0.3148), (**E**) Vimentin IHC detection for either liver (*p* = 0.9818) or tumor (*p* = 0.6011) or β-catenin IHC detection in liver (*p* = 0.0847). Unpaired, two tailed *t*-test. * *p* < 0.05.

**Figure 3:**
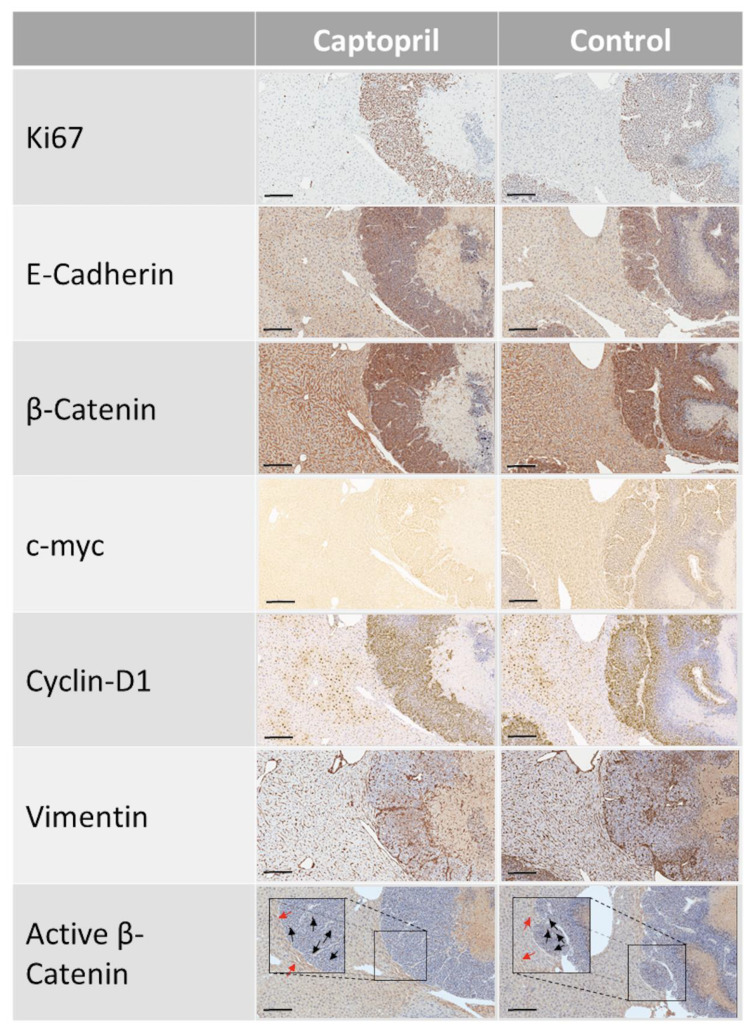
IHC on tissues from captopril treated and control mice. Fixed sections (control *n* = 9, captopril *n* = 10) were stained for Ki67, E-Cadherin, β-catenin, c-myc, cyclin D1, vimentin and active β-catenin. Scale bar, 200 µm.

To investigate the effects of captopril on CRLM in the context of liver regeneration, mice underwent partial hepatectomy 7 days after hepatic metastasis was induced by injecting colorectal cancer cells into the spleen. The expression of the RAS pathway [angiotensin receptor type 1 (AT1R); angiotensin-converting enzyme (ACE), angiotensinogen (AGT)] and Wnt pathway [c-myc, cyclin D1, glutamine synthetase, CD44; Frizzled-7 (Fzd7)] signaling was analyzed by quantitative (q) reverse transcriptase (RT)-PCR on tissues harvested from captopril- and saline-treated mice (Figure 1 and Figure S1). The expression of AT1R, ACE, AGT, glutamine synthetase, CD44 and Fzd7 were not significantly changed in the tumor tissues from the captopril treated mice compared to control mice (Figure 1A–C,F–H, respectively). There were trends towards increased expression of AT1R, ACE and Fzd7 in the tumors of captopril treated mice (Figure 1A,B,H, respectively) but not in the normal liver tissues (Figure S1A,B,H, respectively). Treatment with captopril was associated with a significant down-regulation in c-myc gene expression in mouse tumors following liver resection, compared to control (Figure 1D). Down-regulation of c-myc was confirmed by immunohistochemistry (IHC) staining of the tumors from treated mice compared to control (Figure 2A and Figure 3). The reduction in c-myc gene expression by qRT-PCR was tumor-specific. qRT-PCR analysis of liver tissue from treated mice compared to control revealed no difference in c-myc gene expression (Figure S1D). Further analysis of the tumor IHC staining which compared c-myc staining at the invasive front to the inner layer also showed a trend for reduced c-myc expression, with the strongest trend at the invasive front (*p* = 0.1465) (Figure S2A). 

### 2.2. Captopril Treatment was also Associated with a Significant Down-Regulation of Cyclin D1 Expression

Captopril treatment was also associated with a significant reduction in cyclin D1 gene expression in mouse tumors following liver resection, compared to the control. Treatment was associated with a more than two-fold reduction in cyclin D1 expression by qRT-PCR (Figure 1E). This response was not elicited in the analysis of respective liver tissues and was therefore also tumor-specific (Figure S1E). IHC analysis of cyclin D1 staining revealed no significant reduction in overall cyclin D1 staining (Figure 2C and Figure 3), although there was a slight reduction associated with captopril treatment. When we examined the pattern of IHC staining more closely by comparing the expression of cyclin D1 at the invasive front with the inner layer; captopril treatment was not associated with any significant regional reduction in cyclin D1 expression (Figure S2C).

### 2.3. Captopril-Treated Tumors Maintained Ki67 Expression

Assessment of Ki67 staining, a measure of cells that are in any of the active cell cycle phases [32], revealed that whilst cyclin D1 and c-myc gene expression were significantly down-regulated (Figure 1D,E), and that for c-myc this correlated with a reduction in IHC staining (Figure 2A, Figure S2A and Figure 3), this did not correlate with a reduction in Ki67 staining (Figure 2B, Figure S2B and Figure 3). Tumors of captopril-treated mice maintained their Ki67 positivity despite reduced c-myc IHC staining. The retention of Ki-67 expression (Figure 2B, Figure S2B and Figure 3) is consistent with the sustained expression of E-cadherin and a lack of an EMT phenotype, as dedifferentiated and disseminating CRC tumor cells do not express Ki-67 or E-cadherin [14].

### 2.4. Captopril Treatment was Associated with Significantly Greater Cytoplasmic Staining of β-Catenin

IHC demonstrated that β-catenin staining in the tumors of treated mice, was significantly higher than the control (Figure 2F and Figure 3). Furthermore, β-catenin staining was higher at the invasive front of tumors compared to center from control and treated mice (Figure S2F). The increased β-catenin at the tumor invasive front was paralleled by increased vimentin expression (Figure S2E). β-catenin staining of the tumor tissue was cytoplasmic and nuclear, compared to the liver tissue where staining was overwhelmingly membranous (Figure 3). Cytoplasmic and nuclear staining is a hallmark of active Wnt signaling, since the increased β-catenin in the cytoplasm translocates into the nucleus to drive the transcription of Wnt target genes [16]. The presence of transcriptionally active β-catenin was confirmed by staining with an active β-catenin antibody (Figure 3 and Figure S3) [33]. Increased cytoplasmic β-catenin staining of treated tumors occurs alongside a slight increase in E-cadherin staining (Figure 2D and Figure 3). E-cadherin expression was greater at the invasive front compared to tumor center, while captopril treatment was associated with a trend towards increased E-cadherin staining (Figure S2D and Figure 3). Elevated β-catenin and decreased or absent E-cadherin staining is associated with EMT and tumor cell invasion in CRC [14]. Given that we observed sustained or a trend towards increased E-cadherin expression at the invasive front, these findings, taken together, suggest that captopril treatment leads to increased cell-cell adherens junctions, thereby reducing the tumor invasive phenotype and potential for metastasis.

### 2.5. Captopril Treatment Did Not Significantly Modulate the Expression of Bromodomain and Extra-Terminal Domal Domain (BET) Proteins

Recently, inhibition of BRD4, the chromatin reader, has been shown to specifically reduce the expression of myc in many different cancer types [34,35,36,37,38]. Members of the bromodomain and extra-terminal domain (BET) proteins (BRD2, BRD3, BRD4) modulate gene expression by recruiting transcriptional regulators to specific genome locations. Furthermore, inhibition of BETs has been shown to limit hepatic fibrosis [39] and the BET inhibitor, JQ1, has been shown to block ANGII induced signaling [40]. For this reason, we also investigated the expression of BRD4 to determine whether captopril may be reducing c-myc expression via modulating BET proteins.

**Figure 4:**
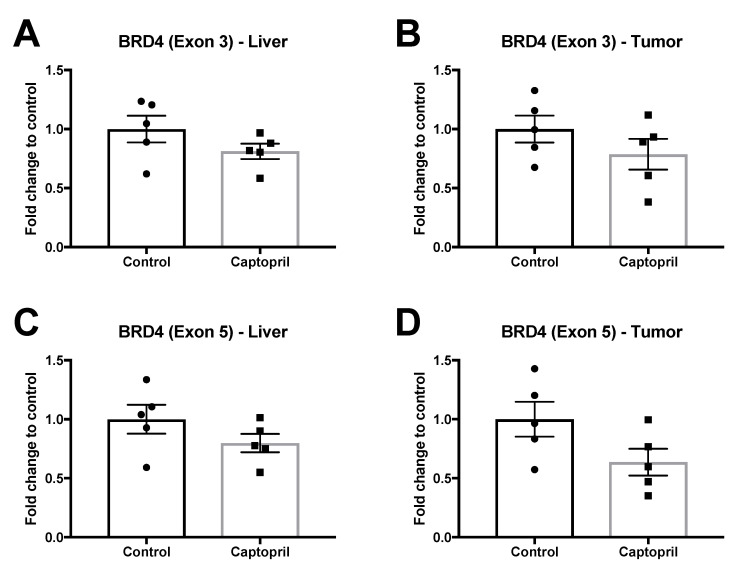
BRD4 expression (qRT-PCR) in liver and tumor tissues. Captopril treatment was not associated with any significant changes in BRD4 expression. (**A**) BRD4 Exon 3 liver (*p* = 0.1843). (**B**) BRD4 Exon 3 tumor (*p* = 0.2533). (**C**) BRD4 Exon 5 liver (*p* = 0.1994). (**D**) BRD4 Exon 5 tumor (*p* = 0.0865). Unpaired, two tailed *t*-test (*n* = 5 mice).

However, qRT-PCR revealed that captopril treatment was not associated with a significant change in BRD4 expression (Figure 4). Although captopril treatment was associated with small reductions in BRD4 expression, and this was more impressive with BRD4–Exon 5 primers, this did not reach significance (Figure 4). These results suggest that other mechanisms may also be involved in reducing c-myc expression.

## 3. Discussion

CRC frequently metastasizes to the liver, and hepatic metastases may be present at the time of diagnosis or develop metachronously [1]. The mainstay of treatment for colorectal liver metastasis is chemotherapy, with or without surgery. Surgery, for those eligible, offers the best chance of cure. However, clinical evidence demonstrates that, following liver resection, many patients develop disease recurrence. The observation that most recurrences occur within six months of surgery has led to research examining the role of post-hepatectomy liver regeneration on the hepatic microenvironment and how this may promote cancer recurrence.

Canonical Wnt signaling regulates stem cell self-renewal, cell proliferation and cell fate decisions. Approximately 80% of all sporadic CRCs occur in association with a mutation in the Wnt/β-catenin signaling pathway. Human colorectal cancers contain multiple mutations affecting the Wnt/β-catenin signaling pathway [41]. Mutations in CRC affecting the Wnt/β-catenin pathway most commonly occur in the *APC* gene, leading to pathogenic expression of Wnt target genes, including c-myc and cyclin D1, as a result of failure to successfully degrade cytoplasmic β-catenin [42]. However, despite constitutive activation of Wnt/β-catenin signaling by mutant *APC*, additional regulation of the Wnt signaling pathway occurs in a spaciotemporal manner, so that bursts of intense Wnt/β-catenin signaling are observed during tumor progression; for example, signaling was found to occur at the invasive front of CRC associated with EMT [16] and during MET to establish the metastases at the secondary site [15]. Tumor invasion and metastasis are underscored by EMT and MET [14,18].

In mouse models, mutant *APC*-induced activation of Wnt/β-catenin signaling and consequent tumor formation is dependent on c-myc, as restoring c-myc in *APC*-mutant tumors abrogates tumor formation and Wnt/β-catenin signaling [43,44]. Indeed, tumors appear to become addicted to myc and withdrawing myc expression has been shown by others to suppress tumor growth [45]. The proto-oncogene *MYC* is tightly regulated in non-cancerous cells due to its oncogenic potential, but is dysregulated in greater than 50% of human cancers [46]. Myc regulates a wide range of cellular processes including signal transduction, cell cycle activity, metabolism, translation, cell adhesion, DNA repair and protein synthesis [46]. Myc has a well-documented role in tumor initiation and more recently its role in tumor maintenance is becoming better understood. 

Our laboratory has previously demonstrated significant tumor reduction in a mouse model of CRLM using RASi captopril or irbesartan [31]. In this study, we explored the effects of captopril treatment on the Wnt/β-catenin pathway and the phenotypic changes associated with tumor progression in the regenerating liver. Our results demonstrate significant reduction in c-myc expression, a recognized target gene of the Wnt/β-catenin signaling pathway [47], in tumors from mice treated with captopril. Down-regulation of c-myc is likely to be one mechanism through which captopril and other RASi are capable of attenuating tumor growth and progression. RASi are commonly used to treat hypertension and cardiac failure; however, they also have a wide range of effects which are not limited to the cardiovascular system [29,48]. RASi have established roles in multiple processes influencing cancer progression, including angiogenesis, extracellular matrix remodeling, cellular proliferation, EMT and immunomodulation [27,29,30,48]. Recently, it has been demonstrated that RAS genes are also down-stream targets of Wnt signaling, and that blocking Wnt signaling down-regulates the expression of RAS genes, reversing the clinical effects of renal fibrosis [24]. This raises the possibility that RASi may influence up-stream functioning of the Wnt pathway.

We showed that captopril treatment, in this CRLM tumor model, was associated with significantly higher levels of cytoplasmic and nuclear β-catenin, a hallmark of active Wnt/β-catenin signaling. Unexpectedly, we observed a significant reduction in the expression of both Wnt target genes, c-myc and cyclin D1, in the captopril-treated tumors. However, several signaling pathways converge on c-myc and cyclin D1, suggesting that in this context, the sum effect is decreased expression. The reduction of c-myc in this study is likely to be highly clinically significant, as we observed a more than two-fold reduction in c-myc mRNA and protein expression in association with treatment. Furthermore, this finding was tumor-specific. Despite myc being discovered over 30 years ago, it has eluded effective drug targeting because rather than being mutated in cancer [49], it is overexpressed due to aberrant upstream signaling or as a result of events which induce amplification of the *myc* gene locus or insertions of activating sequences [50]. Additionally, it has been difficult to target oncogenically up-regulated myc without inhibiting ‘normal’ myc expression, which is required by healthy cells for processes such as cellular proliferation. Importantly, here we demonstrated tumor-specific myc reduction. Also, we reported that mRNA expression of cyclin D1, a key cell cycle checkpoint regulator, was reduced, although this could not be confirmed by IHC.

Despite reductions in both c-myc and cyclin D1 gene expression, Ki67 tumor staining did not change, demonstrating that these tumor cells are actively dividing. This result is consistent with the maintained expression of E-cadherin and Fzd7 we observed in the captopril-treated tumors. Collectively, these findings are consistent with reduced EMT and thus, inhibition of tumor phenotype associated with invasion. EMT in CRC is associated with a loss of E-cadherin and Ki-67 [14] and decreased FZD7 [15]. Notably, Fzd7 expression is decreased at the invasive front of CRCs even though it is a Wnt/β catenin target gene [19,51]. This is one of many examples of context dependent Wnt/β catenin target gene expression. 

Recently, BET proteins have emerged as regulators of myc expression in a variety of tumors [34,38] and this led us to investigate whether captopril treatment modulated BET protein expression in this model. We observed no change in the expression of BRD4 when examining two different exons, suggesting that c-myc modulation in this model is not occurring via BET gene expression.

Although the precise underlying mechanism for c-myc down-regulation remains elusive, we have shown that captopril induced a significant reduction c-myc and cyclin D1 expression. This likely represents an important mode of action for the anti-tumor effects of captopril. The finding that captopril reduced these two very important genes, which have key down-stream effects on the progression of colorectal tumors in vivo, is novel and could have important clinical ramifications for all patients with c-myc-addicted cancers. Indeed, our findings are consistent with observations in leukemia continuous cell lines where captopril also decreased c-myc expression [52]. RASi have been licensed for use in hypertension and cardiac failure for many years and could easily be adapted for the treatment of patients with c-myc-addicted cancers.

## 4. Materials and Methods 

### 4.1. Animal Experiments

Animal experiments were performed following local animal ethics committee (AEC) approval (AEC.05435). Male CBA mice aged 9 weeks were obtained from the Animal Resources Centre in Western Australia. Mice were housed in standard conditions with controlled temperature and humidity, and a strict 12-h light/dark cycle for the duration of the experiments. They received a standard laboratory diet and had access to water at all times. Mice were housed for one week prior to the commencement of experiments in accordance with local AEC policy and experiments commenced when the mice were 10 weeks of age and weighed 20–25 g. On experimental Day 0 mice underwent surgical induction of hepatic metastases via splenic injection of the primary murine colon cancer cell line (MoCR) derived from a dimethyl-hydrazine-induced primary colon carcinoma in the CBA mouse as previously described [28]; on Day 4, intraperitoneal injections of captopril 250 mg/kg commenced and were continued daily for the duration of the experiment. On Day 7, mice underwent a 70% partial hepatectomy and were culled at experimental end-point on Day 16. The induction of colorectal metastases via splenic injection has been published previously [31]. Partial hepatectomy and intraperitoneal injections were carried out according to a previously published methodology [53].

### 4.2. qRT-PCR

Animals were euthanized at experimental end-point, Day 16, following tissue procurement which was carried out under general anesthetic. Livers were flushed with 10 mL of sterile saline prior to procurement to wash out any blood. Liver and colorectal metastases tissues were dissected and separated. Samples were then homogenized using the Qiagen, TissueLyser II in 1 mL of Trizol (Invitrogen) to obtain total RNA. cDNA synthesis and quantitative real-time PCR (qRT-PCR) was performed as previously described [15]. Gene expression levels were calculated relative to the house-keeping gene, β2-microglobulin (b2m) as previously described [21]. Primer sequences are listed in Table S1.

### 4.3. Immunohistochemistry

Formalin-fixed murine tissues obtained from the experiments described above were used for immunohistochemistry (IHC) following paraffin embedding. Sections (4 µm) were cut and mounted on SuperFrost slides (Menzel-Glaser). Antigen retrieval was performed using 1:10 Tris buffer [DAKO Target Retrieval Solution, pH 9 (S2367)], at 99 °C for 30 min. Peroxidase-blocking solution was applied for 30 min (DAKO Peroxidase-Blocking solution S2023). Slides were incubated with 10% normal goat serum. Primary antibodies included vimentin (Abcam, ab92547), Ki67 (Thermo-Scientific SP6), Cyclin D1 (Abcam, ab134175), c-myc (Santa-Cruz, sc-764), E-Cadherin (Santa Cruz, sc-7870), β-Catenin (Santa Cruz, sc-7199) and active β-catenin (Sigma-Aldrich, 05-665). Control sections were incubated with rabbit control antibody. Anti-rabbit horseradish peroxidase secondary antibody was applied for 1 h to all sections (Dako EnVision + System-HRP K4003) and the final detection step was performed using 3,3 diaminobenzidine (Abcam DAB Substrate Kit, ab64238). Antibodies used are listed in Table S2. Slides were scanned on the Monash Histology Platform using the Aperio system. Slides were then analyzed for staining positivity using Aperio ImageScope software. Staining positivity was calculated for the liver and whole tumor tissue portions separately. A further analysis was undertaken to compare the pattern of staining within tumors. The tumor invasive front was defined as the outermost 100 microns of tumor and the tumor inner layer was defined as the 100 microns of tumor tissue interior to this.

### 4.4. Statistics

Statistical analysis was performed using GraphPad Prism using the unpaired student-*t* test or one-way ANOVA and Tukey post hoc test as indicated in the legends; *p* values less than 0.05 were considered significant.

## 5. Conclusions

In conclusion, tumor recurrence following hepatic resection for CRLM and other primary liver cancers remains a major clinical problem for hepatobiliary surgeons. Treatments that can be safely administered alongside surgery to negate this risk may prolong patient survival. The micro-environment of the regenerating liver following partial hepatectomy is unique and many factors may promote tumor growth. The Wnt/β-catenin pathway is just one signaling pathway involved in both tumorigenesis and liver regeneration. Inhibition of this pathway has been shown to modulate EMT and provide a mechanism for reversing both fibrosis and improving cancer outcomes. Here, we have shown that captopril appears to increase Wnt signaling whilst reducing phenotypic features of EMT, providing a mechanism for tumor control in the regenerating liver. We also demonstrated a significant reduction in both c-myc and cyclin D1, although the mechanism is unclear. Whilst we have not elucidated the precise mechanism for c-myc or cyclin D1 down-regulation, these findings have important clinical ramifications. C-myc has been difficult to target therapeutically, so the finding that captopril down-regulates this important proto-oncogene deserves further attention and may provide an alternative therapy for patients with myc addicted cancers.

## Data Availability

Data is contained within the article and supplementary material.

## Supplementary Materials

The following are available online at https://www.mdpi.com/article/10.3390/cancers13112734/s1, Figure S1: Gene expression (qRT-PCR) in liver samples. Figure S2: Spatial IHC staining—invasive front vs inner layer. Figure S3: Active β-Catenin IHC Staining. Table S1: Forward and reverse primers used for qRT-PCR (5’ to 3’). Table S2. List of primary antibodies.
