# Supplementary material for: Captopril, a Renin-Angiotensin System Inhibitor, Attenuates Features of Tumor Invasion and Down-Regulates C-Myc Expression in a Mouse Model of Colorectal Cancer Liver Metastasis
Source: Cancers (Basel). 2021 May 31;13(11):2734. doi: 10.3390/cancers13112734 (PMC8199217; doi:10.3390/cancers13112734)
Supplement: Supplementary file 1 [file cancers-13-02734-s001.zip › cancers-1205419-supplementary.pdf]

Supplementary Materials: Captopril, a Renin-Angiotensin System Inhibitor, Attenuates Features of Tumor Invasion and Down-Regulates C-Myc Expression in a Mouse Model of Colorectal Cancer Liver Metastasis

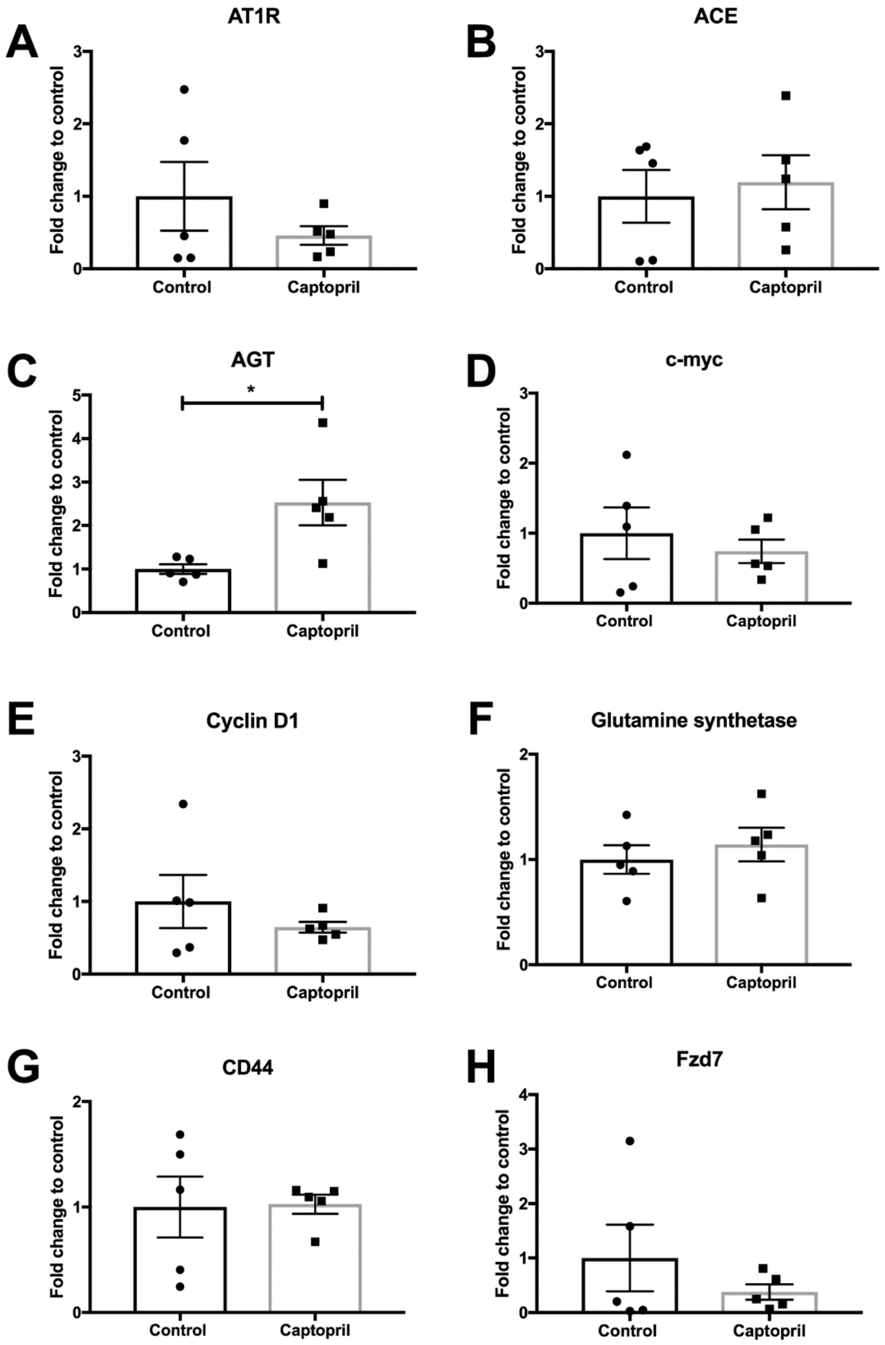

Figure S1. Gene expression (qRT-PCR) in liver samples. Liver tissues from saline (control, n=5) and captopril (n=5) treated mice were analysed by qRT-PCR for the expression of the indicated genes. Captopril treatment significantly increased (C) AGT (p=0.0210) expression. Captopril was not associated with any significant changes in (A) AT1R (p=0.3035), (B)

ACE (p=0.7198), (D) c-myc (0.5394), (E) cyclin-D1 (p=0.3723), (F) glutamine synthetase (p=0.5142), (G) CD44 (p=0.9319) or (H) Fzd7 (p=0.3498) expression in regenerating liver tissue. Un-paired, two tailed T test.

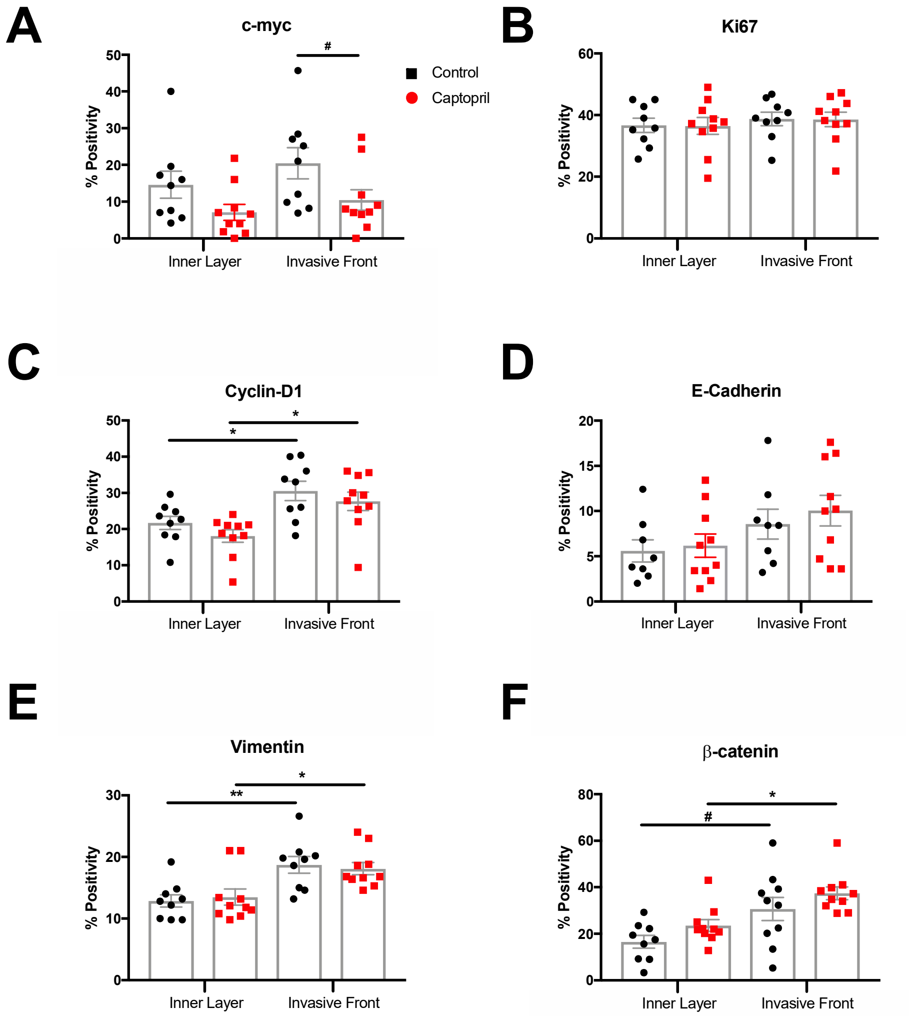

Figure S2. Spatial IHC staining – tumor invasive front vs inner layer. Fixed tissue sections (control, n=9 and captopril, n=10) were stained for the indicated proteins. (A) There was a trend towards decreased c-myc expression in captopril treated tissues compared their respective controls, with the strongest trend at the invasive front (#p=0.1465). (B) Ki67 staining did not change spatially or with captopril treatment. (C) Cyclin D1 expression was greater at the invasive front compared to the inner layer in tissues from control and captopril treated mice (control mice: invasive front vs inner layer *p=0.0453; and captopril treated mice: invasive front vs inner layer *p=0.0176). Captopril treatment did not alter the expression of cyclin D1. (D). E-cadherin expression was greater at the invasive front compared to the inner layer in both control and captopril treated mice. Captopril treatment did not alter the expression of E-Cadherin. (E) Vimentin expression was greater at the invasive front compared to inner layer in both control and captopril treated mice (control mice: invasive front vs inner layer **p=0.0084; and captopril treated mice: invasive front vs inner layer *p=0.036). Captopril treatment did not alter the expression of vimentin. (F) β-catenin expression was greater at the invasive front compared to inner layer in both control and captopril treated mice (control mice: invasive front vs inner layer #p=0.0895; and captopril treated mice: invasive front vs inner layer *p=0.0139). Captopril treatment did not alter the expression of β-catenin. Statistics performed by one-way ANOVA and Tukey post hoc test.

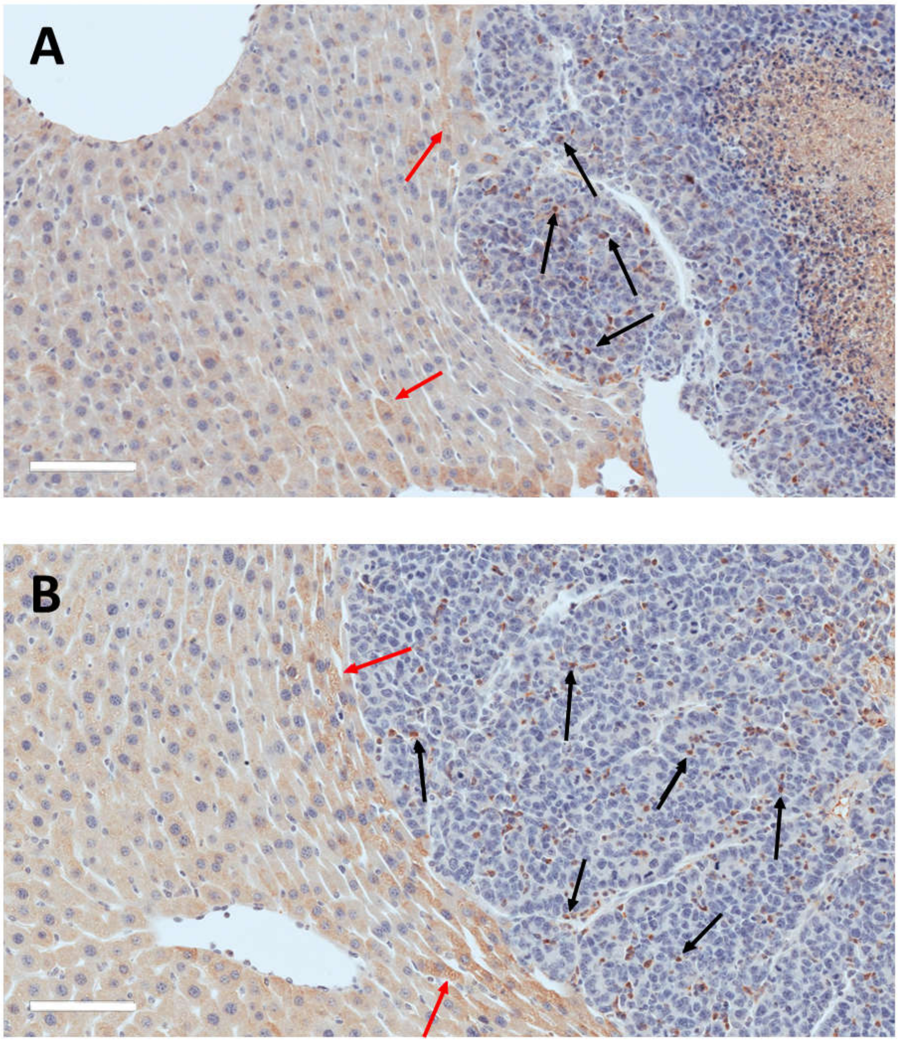

Figure S3. Active β-Catenin IHC Staining. Tissue sections from (A) control and (B) captopril treated mice stained with active β-catenin antibody demonstrating staining in both the tumour and surrounding liver tissues, indicating active Wnt signalling (red arrows indicate cytoplasmic staining, while black arrows indicate nuclear staining). Scale bar, 100 µm.

Table S1. Forward and reverse primers used for qRT-PCR (5′ to 3′).

Gene	Primer			
	**Forward sequence**	**Size (bp)**	**Reverse sequence**	**Size (bp)**
Angiotensin type 1 receptor (AT1R)	GGGCAGTTTATACCGCTATGGA	22	TGGCCGAA-GCGATCTTACAT	20
Angiotensin converting enzyme (ACE)	CAGAATCTACTCCACTGG-CAAGGT	24	TCGTGAGGAAGCCAG-GATGT	20
Angiotensinogen (AGT)	CTGCTCCAGGCTTTCGTCTAA	21	AGAACTGGGTCAGTGGA-TAAATCC	24
c-myc	TAGTGCTGCATGAGGAGACA	20	GGTTT-GCCTCCTCTCCACAG	20
Cyclin-D1	TCGTGGCCTCTAAGATGAAGGA	21	TCGGGCCGGATAGAGTTGT	19

Glutamine synthetase	TTTATCTTGCATCGGGTGTG	20	TTGATGTT-GGAGGTTTCGTG	20
CD44	GTCTGCATCGCGGTCAATAG	20	GGTCTCTGATGGTTCCTT-GTT	21
Fzd7	GCTTCCTAGGTGAGCGTGAC	20	AACCCGACAGGAA-GATGATG	20
β2-microglobulin (b2m)	TTCACCCCCACTGAG	15	GTCTTGGGCTCGGCC	15

Table S2. List of primary antibodies

**Antibody**	**Dilution**
β-Catenin (Santa Cruz, sc-7199)	1:250
c-myc (Santa-Cruz, sc-764)	1:250
Cyclin D1 (Abcam, ab134175)	1:250
E-Cadherin (Santa Cruz, sc-7870)	1:250
Ki67 (Thermo-Scientific SP6)	1:200
Vimentin (Abcam, ab92547)	1:200
Active β-Catenin (Sigma-Aldrich, 05-665)	1:250
